# Quinoxalin-2-yl *o*-tolyl ether

**DOI:** 10.1107/S1600536808026810

**Published:** 2008-08-23

**Authors:** Nor Duha Hassan, Hairul Anuar Tajuddin, Zanariah Abdullah, Seik Weng Ng

**Affiliations:** a Department of Chemistry, University of Malaya, 50603 Kuala Lumpur, Malaysia

## Abstract

The dihedral angle between the two aromatic ring systems in the title compound, C_15_H_12_N_2_O, is 85.9 (1)°; The angle at the O atom is widened to 118.2 (2)°. The quinoxalin­yloxy part of the mol­ecule lies on a mirror plane and the tolyl group is disordered over two positions about the mirror plane.

## Related literature

The title compound exhibits fluorescence; see: Abdullah (2005 ▶); Kawai *et al.* (2001 ▶); Mohd Salleh *et al.* (2007 ▶).
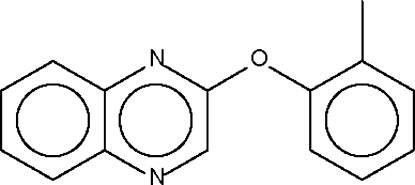


## Experimental

### 

#### Crystal data

C_15_H_12_N_2_O
*M* *_r_* = 236.27
Monoclinic, 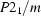

*a* = 7.874 (2) Å
*b* = 7.413 (1) Å
*c* = 10.596 (2) Å
β = 108.332 (3)°
*V* = 587.1 (2) Å^3^
*Z* = 2
Mo *K*α radiation
μ = 0.09 mm^−1^
*T* = 100 (2) K
0.20 × 0.20 × 0.08 mm

#### Data collection

Bruker SMART APEX diffractometer
Absorption correction: none
3392 measured reflections
1443 independent reflections
1032 reflections with *I* > 2σ(*I*)
*R*_int_ = 0.026

#### Refinement

*R*[*F* ^2^ > 2σ(*F* ^2^)] = 0.065
*wR*(*F* ^2^) = 0.183
*S* = 1.03
1443 reflections
119 parameters
H-atom parameters constrained
Δρ_max_ = 0.24 e Å^−3^
Δρ_min_ = −0.31 e Å^−3^

### 

Data collection: *APEX2* (Bruker, 2007 ▶); cell refinement: *SAINT* (Bruker, 2007 ▶); data reduction: *SAINT*; program(s) used to solve structure: *SHELXS97* (Sheldrick, 2008 ▶); program(s) used to refine structure: *SHELXL97* (Sheldrick, 2008 ▶); molecular graphics: *X-SEED* (Barbour, 2001 ▶); software used to prepare material for publication: *publCIF* (Westrip, 2008 ▶).

## Supplementary Material

Crystal structure: contains datablocks global, I. DOI: 10.1107/S1600536808026810/bt2769sup1.cif

Structure factors: contains datablocks I. DOI: 10.1107/S1600536808026810/bt2769Isup2.hkl

Additional supplementary materials:  crystallographic information; 3D view; checkCIF report

## Figures and Tables

**Fig. 1.**
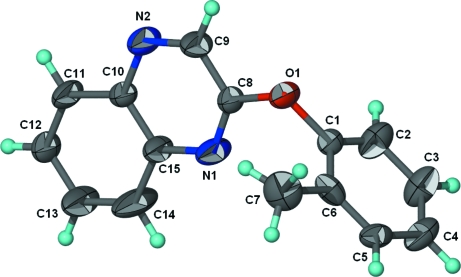
Thermal ellipsoid plot (Barbour, 2001) of C15H12N2O at the 70% probability level; hydrogen atoms are drawn as spheres of arbitrary radius.

## supplementary crystallographic information

### Comment

(type here to add)

### Experimental

*o*-Cresol (0.54 g, 5 mmol) was dissolved in a small volume of water containing potassium hydroxide (0.20 g, 5 mmol). The mixture was heated to remove the water to give a brown compound. The compound and 2-chloroquinoxaline (0.82, g, 5 mmol) were heated in THF (15 ml) for 8 h. The mixture was in 1 N sodium hydroxide; the aqueous solution was extracted with dichloromethane. The organic phase was dried over sodium sulfate. Evaporation of the solvent gave a yellow product, which was was washed with chloroform to remove impurities. Crystals were obtained upon recrystallization from an ethyl acetate/hexane mixture.

### Refinement

The tolyl group is disordered about a mirror plane; the phenylene ring was refined as a rigid hexagon. The seven carbon atoms were allowed to refine off the symmetry element; the atoms were all given 0.5 site occupancy.

H-atoms were placed in calculated positions (C—H 0.95–0.98 Å) and were included in the refinement in the riding model approximation, with *U*(H) fixed at 1.2–1.5*U*(C).

### Crystal data

**Table:**

C_15_H_12_N_2_O	*F*_000_ = 248
*M**_r_* = 236.27	*D*_x_ = 1.337 Mg m^−^^3^
Monoclinic, *P*2_1_/*m*	Mo *K*α radiation λ = 0.71073 Å
Hall symbol: -P 2yb	Cell parameters from 756 reflections
*a* = 7.874 (2) Å	θ = 2.7–24.6º
*b* = 7.413 (1) Å	µ = 0.09 mm^−^^1^
*c* = 10.596 (2) Å	*T* = 100 (2) K
β = 108.332 (3)º	Block, colorless
*V* = 587.1 (2) Å^3^	0.20 × 0.20 × 0.08 mm
*Z* = 2	

### Data collection

**Table:**

Bruker SMART APEX diffractometer	1032 reflections with *I* > 2σ(*I*)
Radiation source: fine-focus sealed tube	*R*_int_ = 0.027
Monochromator: graphite	θ_max_ = 27.5º
*T* = 100(2) K	θ_min_ = 2.0º
ω scans	*h* = −10→8
Absorption correction: None	*k* = −9→8
3392 measured reflections	*l* = −12→13
1443 independent reflections	

### Refinement

**Table:**

Refinement on *F*^2^	Secondary atom site location: difference Fourier map
Least-squares matrix: full	Hydrogen site location: inferred from neighbouring sites
*R*[*F*^2^ > 2σ(*F*^2^)] = 0.065	H-atom parameters constrained
*wR*(*F*^2^) = 0.183	*w* = 1/[σ^2^(*F*_o_^2^) + (0.0776*P*)^2^ + 0.4106*P*] where *P* = (*F*_o_^2^ + 2*F*_c_^2^)/3
*S* = 1.03	(Δ/σ)_max_ = 0.001
1443 reflections	Δρ_max_ = 0.24 e Å^−^^3^
119 parameters	Δρ_min_ = −0.31 e Å^−^^3^
Primary atom site location: structure-invariant direct methods	Extinction correction: none

### Fractional atomic coordinates and isotropic or equivalent isotropic displacement parameters (Å^2^)

**Table:**

	*x*	*y*	*z*	*U*_iso_*/*U*_eq_	Occ. (<1)
O1	0.1778 (2)	0.2500	0.64890 (18)	0.0405 (6)	
N1	0.4752 (3)	0.2500	0.7791 (2)	0.0552 (9)	
N2	0.3518 (3)	0.2500	0.9997 (2)	0.0479 (8)	
C1	0.2266 (3)	0.1943 (3)	0.5368 (2)	0.0256 (8)	0.50
C2	0.2256 (5)	0.0171 (3)	0.4936 (3)	0.047 (2)	0.50
H2	0.2002	−0.0787	0.5445	0.056*	0.50
C3	0.2619 (5)	−0.0199 (3)	0.3761 (3)	0.0473 (11)	0.50
H3	0.2612	−0.1410	0.3466	0.057*	0.50
C4	0.2991 (4)	0.1203 (4)	0.3017 (2)	0.0417 (12)	0.50
H4	0.3239	0.0950	0.2213	0.050*	0.50
C5	0.3000 (3)	0.2975 (3)	0.3449 (2)	0.0345 (15)	0.50
H5	0.3255	0.3933	0.2940	0.041*	0.50
C6	0.2638 (3)	0.3345 (3)	0.4624 (3)	0.0313 (8)	0.50
C7	0.2599 (11)	0.5228 (12)	0.5056 (7)	0.0496 (17)	0.50
H7A	0.3449	0.5946	0.4761	0.074*	0.50
H7B	0.2932	0.5272	0.6028	0.074*	0.50
H7C	0.1391	0.5719	0.4666	0.074*	0.50
C8	0.3083 (3)	0.2500	0.7686 (2)	0.0300 (6)	
C9	0.2449 (4)	0.2500	0.8784 (3)	0.0393 (8)	
H9	0.1194	0.2500	0.8633	0.047*	
C10	0.5314 (3)	0.2500	1.0167 (2)	0.0284 (6)	
C11	0.6553 (4)	0.2500	1.1448 (3)	0.0429 (8)	
H11	0.6135	0.2500	1.2197	0.051*	
C12	0.8343 (4)	0.2500	1.1638 (3)	0.0398 (8)	
H12	0.9169	0.2500	1.2513	0.048*	
C13	0.8952 (4)	0.2500	1.0547 (3)	0.0631 (12)	
H13	1.0201	0.2500	1.0674	0.076*	
C14	0.7769 (4)	0.2500	0.9289 (3)	0.0889 (19)	
H14	0.8209	0.2500	0.8551	0.107*	
C15	0.5922 (3)	0.2500	0.9066 (3)	0.0378 (7)	

### Atomic displacement parameters (Å^2^)

**Table:**

	*U*^11^	*U*^22^	*U*^33^	*U*^12^	*U*^13^	*U*^23^
O1	0.0199 (9)	0.0771 (16)	0.0236 (9)	0.000	0.0055 (7)	0.000
N1	0.0196 (11)	0.123 (3)	0.0224 (12)	0.000	0.0065 (9)	0.000
N2	0.0248 (12)	0.093 (2)	0.0270 (12)	0.000	0.0090 (9)	0.000
C1	0.0198 (14)	0.033 (2)	0.0229 (16)	0.0038 (12)	0.0051 (12)	0.0008 (12)
C2	0.057 (4)	0.056 (5)	0.031 (3)	0.004 (3)	0.018 (3)	0.002 (3)
C3	0.072 (3)	0.041 (3)	0.032 (2)	0.015 (2)	0.021 (2)	0.0047 (19)
C4	0.040 (2)	0.064 (4)	0.024 (2)	0.015 (2)	0.0142 (17)	0.002 (2)
C5	0.0231 (15)	0.054 (5)	0.0263 (17)	−0.0015 (15)	0.0075 (13)	0.0121 (16)
C6	0.0175 (15)	0.0301 (19)	0.039 (2)	−0.0008 (15)	−0.0015 (15)	0.0020 (17)
C7	0.055 (3)	0.037 (3)	0.054 (4)	−0.001 (3)	0.013 (3)	0.008 (3)
C8	0.0220 (12)	0.0435 (16)	0.0233 (12)	0.000	0.0053 (10)	0.000
C9	0.0188 (12)	0.069 (2)	0.0304 (14)	0.000	0.0084 (11)	0.000
C10	0.0230 (12)	0.0369 (15)	0.0259 (13)	0.000	0.0086 (10)	0.000
C11	0.0303 (14)	0.076 (2)	0.0227 (13)	0.000	0.0093 (11)	0.000
C12	0.0265 (14)	0.065 (2)	0.0243 (13)	0.000	0.0020 (11)	0.000
C13	0.0211 (14)	0.137 (4)	0.0291 (15)	0.000	0.0053 (12)	0.000
C14	0.0238 (15)	0.221 (6)	0.0247 (16)	0.000	0.0111 (13)	0.000
C15	0.0218 (13)	0.067 (2)	0.0236 (13)	0.000	0.0063 (10)	0.000

### Geometric parameters (Å, °)

**Table:**

O1—C8	1.359 (3)	C6—C7	1.472 (9)
O1—C1	1.420 (3)	C7—H7A	0.9800
O1—C1^i^	1.420 (3)	C7—H7B	0.9800
N1—C8	1.283 (3)	C7—H7C	0.9800
N1—C15	1.375 (3)	C8—C9	1.402 (4)
N2—C9	1.296 (4)	C9—H9	0.9500
N2—C10	1.369 (3)	C10—C15	1.392 (4)
C1—C2	1.3900	C10—C11	1.400 (4)
C1—C6	1.3900	C11—C12	1.360 (4)
C2—C3	1.3900	C11—H11	0.9500
C2—H2	0.9500	C12—C13	1.384 (4)
C3—C4	1.3900	C12—H12	0.9500
C3—H3	0.9500	C13—C14	1.365 (4)
C4—C5	1.3900	C13—H13	0.9500
C4—H4	0.9500	C14—C15	1.398 (4)
C5—C6	1.3900	C14—H14	0.9500
C5—H5	0.9500		
			
C8—O1—C1	117.19 (19)	O1—C8—C9	114.4 (2)
C8—N1—C15	115.8 (2)	N2—C9—C8	122.2 (2)
C9—N2—C10	116.9 (2)	N2—C9—H9	118.9
C2—C1—C6	120.0	C8—C9—H9	118.9
C2—C1—O1	125.13 (17)	N2—C10—C15	120.2 (2)
C6—C1—O1	114.68 (17)	N2—C10—C11	120.2 (2)
C1—C2—C3	120.0	C15—C10—C11	119.6 (2)
C1—C2—H2	120.0	C12—C11—C10	121.2 (3)
C3—C2—H2	120.0	C12—C11—H11	119.4
C2—C3—C4	120.0	C10—C11—H11	119.4
C2—C3—H3	120.0	C11—C12—C13	119.4 (3)
C4—C3—H3	120.0	C11—C12—H12	120.3
C5—C4—C3	120.0	C13—C12—H12	120.3
C5—C4—H4	120.0	C14—C13—C12	120.4 (3)
C3—C4—H4	120.0	C14—C13—H13	119.8
C4—C5—C6	120.0	C12—C13—H13	119.8
C4—C5—H5	120.0	C13—C14—C15	121.3 (3)
C6—C5—H5	120.0	C13—C14—H14	119.4
C5—C6—C1	120.0	C15—C14—H14	119.4
C5—C6—C7	119.7 (3)	N1—C15—C10	121.5 (2)
C1—C6—C7	120.3 (3)	N1—C15—C14	120.4 (2)
N1—C8—O1	122.3 (2)	C10—C15—C14	118.1 (3)
N1—C8—C9	123.4 (2)		
			
C8—O1—C1—C2	87.53 (18)	C1^i^—O1—C8—C9	160.91 (12)
C1^i^—O1—C1—C2	−173.48 (14)	C10—N2—C9—C8	0.000 (2)
C8—O1—C1—C6	−97.56 (16)	N1—C8—C9—N2	0.000 (2)
C1^i^—O1—C1—C6	1.43 (19)	O1—C8—C9—N2	180.000 (1)
C6—C1—C2—C3	0.0	C9—N2—C10—C15	0.000 (2)
O1—C1—C2—C3	174.7 (2)	C9—N2—C10—C11	180.000 (2)
C1—C2—C3—C4	0.0	N2—C10—C11—C12	180.000 (2)
C2—C3—C4—C5	0.0	C15—C10—C11—C12	0.000 (2)
C3—C4—C5—C6	0.0	C10—C11—C12—C13	0.000 (2)
C4—C5—C6—C1	0.0	C11—C12—C13—C14	0.000 (2)
C4—C5—C6—C7	−178.3 (4)	C12—C13—C14—C15	0.000 (2)
C2—C1—C6—C5	0.0	C8—N1—C15—C10	0.000 (1)
O1—C1—C6—C5	−175.2 (2)	C8—N1—C15—C14	180.000 (1)
C2—C1—C6—C7	178.2 (4)	N2—C10—C15—N1	0.000 (2)
O1—C1—C6—C7	3.0 (4)	C11—C10—C15—N1	180.000 (1)
C15—N1—C8—O1	180.000 (1)	N2—C10—C15—C14	180.000 (2)
C15—N1—C8—C9	0.000 (1)	C11—C10—C15—C14	0.000 (2)
C1—O1—C8—N1	19.09 (12)	C13—C14—C15—N1	180.000 (2)
C1^i^—O1—C8—N1	−19.09 (12)	C13—C14—C15—C10	0.000 (2)
C1—O1—C8—C9	−160.91 (12)		

Symmetry codes: (i) *x*, −*y*+1/2, *z*.
